# Population pharmacokinetic data analysis of three phase I studies of matuzumab, a humanised anti-EGFR monoclonal antibody in clinical cancer development

**DOI:** 10.1038/sj.bjc.6604265

**Published:** 2008-03-04

**Authors:** K Kuester, A Kovar, C Lüpfert, B Brockhaus, C Kloft

**Affiliations:** 1 Department of Clinical Pharmacy, Institute of Pharmacy, Freie Universitaet Berlin, Berlin, Germany; 2 Department of Human Pharmacology, Merck KGaA, Darmstadt, Germany; 3 Department of Clinical Pharmacy, Institute of Pharmacy, Martin-Luther-Universitaet Halle-Wittenberg, Halle, Germany

**Keywords:** matuzumab, population pharmacokinetic, monoclonal antibody, linear and nonlinear clearance

## Abstract

A population pharmacokinetic model based on data from three phase I studies was to be developed including a covariate analysis to describe the concentration–time profiles of matuzumab, a novel humanised monoclonal antibody. Matuzumab was administered as multiple 1 h i.v. infusions with 11 different dosing regimens ranging from 400 to 2000 mg, q1w–q3w. For analysis, 90 patients with 1256 serum concentration–time data were simultaneously fitted using the software NONMEM™. Data were best described using a two-compartment model with the parameters central (*V*_1_) and peripheral distribution volume (*V*_2_), intercompartmental (*Q*) and linear (CLL) clearance and an additional nonlinear elimination pathway (*K*_m_, *V*_max_). Structural parameters were in agreement with immunoglobulin characteristics. In total, interindividual variability on *V*_max_, CLL, *V*_1_ and *V*_2_ and interoccasion variability on CLL was 22–62% CV. A covariate analysis identified weight having an influence on *V*_1_ (+0.44% per kg) and CLL (+0.87% per kg). All parameters were estimated with good precision (RSE<39%). A robust population pharmacokinetic model for matuzumab was developed, including a nonlinear pharmacokinetic process. In addition, relevant and plausible covariates were identified and incorporated into the model. When correlated to efficacy, this model could serve as a tool to guide dose selection for this ‘targeted’ cancer therapy.

Recent discoveries in biomolecular processes in tumour growth led to the evolution of new targeted therapies in cancer treatment (Ross *et al*, 2003). Monoclonal antibodies as one of these new potential therapeutic agents benefit from their ability to bind to specific structural targets leading to good clinical efficacy and a general lower incidence of adverse events than small molecule therapeutics (Stern and Herrmann, 2005).

Matuzumab is a humanised recombinant monoclonal antibody (mAb) of the immunoglobulin subclass IgG1 (*κ*-chain) targeting the epidermal growth factor receptor (EGFR, HER1 and c-ErbB-1) (Kollmannsberger *et al*, 2006). This physiological transmembrane receptor with protein tyrosine kinase activity is activated by natural ligands such as epidermal growth factor and transforming growth factor-*α* (Watanabe *et al*, 1994; Wells, 1999; Harari, 2004). The EGFR is constitutively expressed in many healthy epithelial tissues, including skin and hair follicle. It is overexpressed or upregulated in a variety of tumour entities (e.g. colon, mamma and bronchial carcinoma) and is often associated with a high metastatic rate, poor prognosis and advanced disease progression (Dassonville *et al*, 1993; Rusch *et al*, 1993; Salomon *et al*, 1995). Epidermal growth factor receptor can be used as a target for therapies based on blockade of receptor–ligand interactions and inhibition of downstream signalling pathways such as cell proliferation, angiogenesis and invasion as well as increase of apoptosis (Ritter and Arteaga, 2003; Li *et al*, 2005). Epidermal growth factor receptor and other members of this receptor family have already been successful targets for cancer therapy (Wells, 1999).

The history of targeting the EGFR with matuzumab started with the murine mAb 425 generated by immunisation of mice (Murthy *et al*, 1987). From a subclone, the antibody EMD55900 was produced causing immune reactions in patients by developing human anti-mouse antibodies.

The first approved mAb targeting EGFR as a single agent or in combination with irinotecan in patients with EGFR-expressing, metastatic and irinotecan-refractory colorectal carcinoma is the chimeric mAb cetuximab (Harding and Burtness, 2005), which has shown favourable efficacy (Cunningham *et al*, 2004).

The most common adverse reaction was reported to be skin toxicity, including acneform rash. To overcome the disadvantages of the murine or chimeric mAbs and to not or only marginally induce anti-antibodies and cause an immune response, the humanisation of the murine mAb 425 was performed, according to the method of G. Winter and as described by Kettleborough *et al* (1991). The humanised mAb matuzumab has already shown promising activity in several phase I and phase II studies in the treatment of different tumour entities (Vanhoefer *et al*, 2004).

The overall aim of this population analysis was to develop a PK model comprehensively characterising the pharmacokinetics of matuzumab. An accurate description of the typical PK profile and of different variability types over a broad range of dosing regimens was to be provided. In addition, patient-specific characteristics should be attempted to be identified to explain the variability of the PK parameters and to further guide dosage regimen decisions for following trials.

## MATERIALS AND METHODS

### Patient population, treatment and data collection

This study included 90 patients (53 males and 37 females) from three phase I, open labelled, nonrandomised, uncontrolled, multicentre studies. The patients had different types of advanced carcinoma. The main criteria for inclusion encompassed histologically proven carcinoma in advanced, nonresectable and/or metastatic state, Karnofsky performance status ⩾60%, life expectancy >12 weeks (1 study >8 weeks), no prior chemo- or radiotherapy within the last 3 or 4 weeks prior to study start, adequate renal, haematological and hepatic function, age ⩾18 years. Patients were excluded if they had known brain metastases, were pregnant or had other relevant medical criteria. All patients gave written informed consent prior to inclusion into the study. The clinical studies were carried out after approval from ethics committee.

Patients received matuzumab as multiple 1 h i.v. infusions in a constant dosing regimen. A wide range of dosing regimens was covered by different groups of patients. In study 1, matuzumab was administered starting with 400 mg weekly, followed by 800 mg biweekly and up to 800 mg every week in combination with a fixed dose of gemcitabine (1000 mg m^−2^). Study 2 included the administration of 400, 800, 1200 and 1600 mg with a 3-week interval and, additionally, 1200 mg biweekly and weekly administration. In study 3, matuzumab was given 400, 800, 1200 or 1600 mg once weekly. Some patients were treated over a longer time period of approximately 1 year.

Serum samples were taken pre- and post-infusions. Frequent serum samples were taken after the first infusion and after the infusion given 3 weeks later. Before and after several other infusions peak and trough concentrations were analysed. Details of dose regimens and sampling schedules are presented in Table 1. Frozen samples were shipped to the Institute of Drug Metabolism and Pharmacokinetics, Merck KGaA, Grafing, Germany, for bioanalysis. Serum levels were evaluated using a validated sandwich enzyme-linked immunosorbent assay method, as described previously (Vanhoefer *et al*, 2004). Precision and accuracy met the international recommendations for bioanalytical immunoassays (DeSilva *et al*, 2003).

### Population pharmacokinetic analysis

The population PK analysis was performed with serum mAb concentration data and associated data such as demographics from all three studies. The population PK model building process was performed using the nonlinear mixed-effects modelling approach implemented in the software NONMEM™, version V, level 1.1. Model development was performed stepwise. First a structural model was developed including an investigation of different compartment models. During model refinement, linear and nonlinear processes and elimination pathways were included.

During the development of the base model and at later stages, interindividual, residual and interoccasion variabilities were investigated. As part of the statistical submodel, the interindividual variability (IIV) was modelled with an exponential random effects term: 
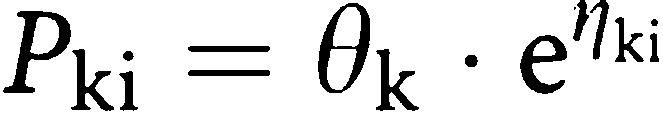


where *P*_ki_ represents the parameter value *k* from the individual i and *θ*_k_ describes the population value of the parameter *k*. *η*_ki_ denotes the ln-difference between *P*_ki_ and *θ*_k_. The interoccasion variability (IOV), that is the variability within one individual between study occasions, was also examined with an exponential random effects term: 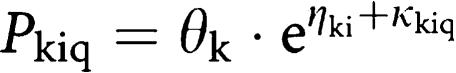


where *P*_kiq_ is the individual parameter value *k* from the individual i at the occasion q that differs from the typical individual value by an additional random effect *κ*_kiq_. An occasion was characterised as the time period from the start of an infusion and until the start of the next administration. *η*_ki_ and *κ*_kiq_ were assumed to be symmetrically distributed with a zero mean and a variance of *ω*^2^ and *π*^2^, respectively.

Residual variability represents the discrepancy between the observed and the model-predicted concentrations after incorporation of IIV and IOV. It was modelled using additive, proportional or combined error models.

All models were parameterised in terms of clearance(s) and volume(s) using the subroutine ADVAN6 TRANS1 TOL5 in NONMEM. The analyses were performed using different estimation methods (first-order, FO; first-order conditional estimation, FOCE, with or without interaction). For the final model, FOCE with interaction was used.

### Covariate model building

To advance the population analysis, a covariate analysis was performed starting from the base model. The analysis included two sequential methods to investigate whether covariates could explain part of the variability of the PK parameters: the GAM analysis (‘generalised additive modelling’, implemented in the software Xpose®), using the Akaike's Information Criterion (Jonsson and Karlsson, 1999; Pan, 2001), and the covariate analysis within NONMEM. The GAM analysis allowed a fast initial screening of covariates (Mandema *et al*, 1992), which were retrieved by the insertion of covariate relations into the NONMEM model. In NONMEM, all covariate relations were investigated by forward inclusion and backward deletion techniques. The final covariate model was built in a stepwise manner. In each step, all possible/remaining parameter–covariate relations were assessed. The included covariate relation that led to the largest drop in the objective function value (OFV), the criterion for model evaluation, was kept in the model. In the next steps, the remaining covariate relations were investigated. All covariate relations that caused a ΔOFV>−3.84 (*P*<0.05, df=1) formed the full covariate model. From the full model, the covariate relations were then deleted one at a time using a stricter criterion (ΔOFV>10.83, *P*<0.001, df=1). The final covariate model was achieved when deletion of each covariate relation was significant.

Covariates investigated for their influence on PK parameters included continuous characteristics as demographics (weight (WT), height (HT), age, body surface area (BSA), body mass index (BMI)), laboratory values (creatinine clearance (CLCR), lactate dehydrogenase (LDH), alkaline phosphatase (AP), white blood cell count (WBC)) and others (Karnofsky index, dose group (DSG)), as well as the categorical characteristics (sex, study number, study site (SID) and concomitant chemotherapy (COME)).

Continuous covariates were investigated with a linear covariate model: 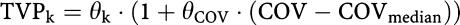


where TVP_k_ is the population value of the parameter *P*_k_ for a specific covariate value (COV) and *θ*_k_ is the population value of the parameter *P*_k_ with the covariate value being the median value (COV_median_). *θ*_cov_ is the fractional change in the population parameter with each unit change from the median covariate value. Whether graphical inspection suggested, nonlinear relations were investigated with a power model, an *E*_max_ model or an exponential model.

Categorical covariates were given as dichotomous variables (sex: male/female, concomitant chemotherapy: yes/no). The coding will be illustrated using an indicator variable (IND), being 0 or 1 (e.g. male or female). 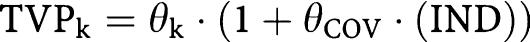


where *θ*_k_ is the typical value of the parameter *P*_k_ when IND=0. *θ*_cov_ represents the fractional increase or decrease of the parameter *P*_k_ caused by IND=1.

If the categorical covariate had multiple categories, each category had an IND (e.g. study site: site 1=(IND) 1, etc.). 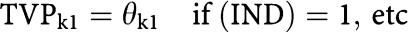


where *θ*_kx_ is the typical value of the parameter *P*_k_ for each category_x_.

Visual exploratory analysis of the covariate relations revealed that for the laboratory parameters aspartate aminotransferase, alanine aminotransferase, gamma glutamyltransferase and bilirubin, a ‘relation’ was driven by very few individuals. Neglecting these individuals did not support the relation anymore. Consequently, these relations were not considered in further covariate analysis.

## RESULTS

### Patient population

Patient characteristics with descriptive statistics are presented in Table 2. For all parameters, the range was wide, for example, age was varying from 29 to 82 years, which was beneficial for the ability to identify covariate relations. The table also includes the number of the missing values that were replaced for the model-building process by the respective median value.

### Base model

**Figure 1:**
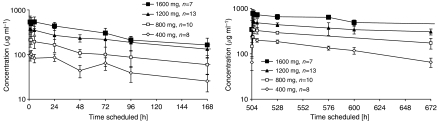
Semilogarithmic plot of the geometric mean and the standard deviation of the observed concentration–time profiles of the four weekly dose regimens (400, 800, 1200 and 1600 mg per week) after the first (left panel) and fourth (right panel) infusion. *n*=number of patients in the dose group. Last time point for 1600 mg dose group was after 1008 h and is not shown.

A total of 1256 serum concentrations from 0.258 to 1157 *μ*g ml^−1^ were simultaneously analysed. The average number of concentrations per patient was 15 (range: 5–24). Serum concentration–time profiles were best described by a two-compartment model (e.g. ΔOFV>−300 compared with a one-compartment model, *P*<0.05). Within this model in addition to the linear clearance (CLL), a second elimination pathway as a nonlinear process (Michaelis–Menten kinetics, CLNL) from the central compartment was included with the additional parameters *V*_max_, the maximum elimination rate (mg h^−1^), and *K*_m_, the concentration (*μ*g ml^−1^) with half-maximal elimination rate (ΔOFV>−100). A need to incorporate nonlinearity might also be concluded from the semilogarithmic plots in Figure 1, showing the geometric mean and the standard deviation of the observed concentration–time profiles of four weekly dose regimens of 400–1600 mg, after the first and fourth infusion. In the terminal phase, the slope of the curve was steeper at lower concentrations.

**Figure 2:**
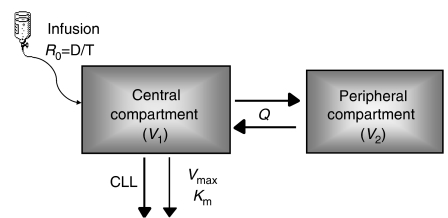
Schematic structural pharmacokinetic model. *R*_0_=infusion rate; *D*=dose; *T*=infusion duration; *V*_1_=volume in the central compartment; *Q*=intercompartmental clearance; *V*_2_=volume in the peripheral compartment; CLL=linear clearance part; *V*_max_=maximum elimination rate; *K*_m_=concentration at which the elimination rate is 50% of the maximum value.

The final base structural model is shown in Figure 2. In this model, the input was the route of administration (i.v. infusion). The central compartment with the central volume of distribution (*V*_1_) was linked with the peripheral compartment with the peripheral volume of distribution (*V*_2_) via *Q*, the intercompartmental clearance. Implementation of CLNL from the peripheral compartment only or from both compartments did not result in an improvement of the model. The linear part of clearance was estimated to be 14.6 ml h^−1^ and the nonlinear part, calculated from the parameters *K*_m_ (5.3 mg l^−1^) and *V*_max_ (0.552 mg h^−1^) at mAb concentrations ≪*K*_m_, to be 104.2 ml h^−1^, respectively.

**Figure 3:**
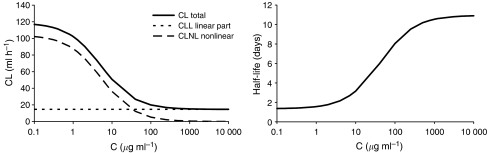
Left panel: dependence of clearance on concentration (*C*) of the mAb. Right panel: dependence of half-life (in days) on *C*.

Total clearance as the sum of CLL and CLNL was 118.8 ml h^−1^ (at mAb concentrations ≪*K*_m_). In Figure 3 left panel, the dependence of total clearance on the concentration of the mAb is presented. At low mAb concentrations, until approximately 1 *μ*g ml^−1^, total clearance (solid line) was mainly influenced by the nonlinear clearance part (long-dashed line). At higher mAb concentrations, the impact of the nonlinear part on the total clearance decreased and the linear part (short-dashed line) was dominating. In accordance with the nonlinear behaviour, the half-life ranged between 4.4 and 10.5 days at concentrations of 20 and 1000 *μ*g ml^−1^, respectively (Figure 3, right panel).

Interindividual variability was quantified for CLL, *V*_1_, *V*_2_ and *V*_max_, and in addition, IOV was implemented. The inclusion of IOV was limited to eight infusions due to insufficient data hereafter and implemented by different ways of assigning the eight infusions to a varying number of occasions. The best result (lowest OFV, smallest relative standard errors in % (RSE=standard error divided by population estimate^*^100)) was achieved with IOV on CLL, where every infusion corresponded to one occasion (ΔOFV∼−400).

In general, in the base model, IIV was moderate (24–60% CV) and larger than IOV of 23% CV. Residual variability was best implemented with a combined error model with a proportional error of 13% CV and an additive error of 0.312 mg l^−1^, being fixed due to model stability (the value was chosen from prior plausible successfully run models).

The parameters obtained from the base model are shown in Table 3, left part, including the RSE. All parameters were generally estimated with good precision (RSE⩽37.1%, except for IIV on CLL and *V*_max_ ⩽49.4%).

### Final model

Fourteen covariate relations were found by GAM: *V*_max_∼BSA, sex, CLCR; *V*_1_∼BSA, COME, sex; *V*_2_∼COME, sex, LDH, SID; CLL∼BSA, COME, AP, sex.

These screening results were included into the NONMEM base model. After forward inclusion, the full covariate model contained eight relations: *V*_max_∼BSA; *V*_1_∼WT, DSG, COME; *V*_2_∼BSA, DSG, COME; CLL∼BSA (ΔOFV in total: −102). After backward elimination, the remaining statistically significant covariate relations were *V*_1_∼WT, DSG, *V*_2_∼COME and CLL∼BSA. The OFV compared with the base model was reduced by 69.

The significant relations identified by NONMEM were further examined for plausibility and relevance. Body size measures were identified to be plausible explanatory factors for the PK parameters *V*_1_ and CLL. Regarding the influence of DSG on *V*_1_, the graphical inspection indicated that the relation was driven by very few patients only in the highest dose group. In addition, the influence of the dose group was inspected by simulation of ‘dummy’ patients of the lowest-, median- and highest-dose group. As the resulting concentration–time profiles were very similar, this covariate relation was removed from the model. An analogous procedure was performed for the covariate COME on CLL. No major difference was found, and, as additionally, no biologically plausible explanation can be given for this covariate relation, it was also removed from the model.

During covariate modelling, IIV on *V*_max_ decreased from the base to the final covariate model, although no covariate on *V*_max_ was included. Therefore, different correlations were investigated revealing correlations between *V*_max_, *V*_1_ and *V*_2_. The correlation coefficients are given in Table 3 (right panel). After the inclusion of the correlations, the model contained the remaining covariates WT on *V*_1_ and BSA on CLL. As BSA and WT were highly correlated, it was examined whether the covariate BSA could be exchanged by WT. It was shown that the model with WT on CLL was not inferior to the model with BSA on CLL. From the individual plots (observed and predicted concentrations *vs* time), no difference between the two covariate models was seen. Additionally, compared with WT, BSA displayed a relatively small range of values, and especially there were only few study patients with high BSA values. Another criterion to support the replacement of the covariates was given by the inspection of the distribution of the individual CLL. The model with WT on CLL better followed a normal distribution pattern. The exchange of the covariates caused an increase in the OFV by only 3.4. As WT is a directly measured variable in the daily clinical process compared with the derived variable BSA, and because, in summary, it was demonstrated that similar results were obtained from the covariate exchange, the final model included WT on CLL instead of BSA, besides WT on *V*_1_. A deviation of −19% to +25% of the population PK parameters (*V*_1_, CLL) for the 5th and 95th percentile of the WT values in the study population with respect to the population PK parameters for the median WT value was observed.

All parameter estimates obtained from the final model are shown in Table 3 (right part). *V*_1_, *V*_2_, CLL, *Q*, *V*_max_ and *K*_m_ of the final model were similar to the estimates of the base model. IIV on CLL and *V*_1_ decreased by 25 and 10%, respectively. In general, all parameters were estimated with a better precision than in the base model (RSE<39%). Especially for the IIV (except on *V*_1_) and IOV, more precise estimates were obtained.

**Figure 4:**
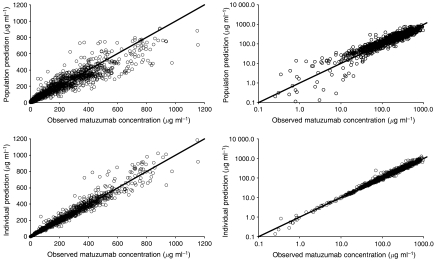
Goodness-of-fit plots. Population predictions (upper panel) and individual predictions (lower panel) *vs* observed matuzumab serum concentrations are shown using linear (left) and logarithmic (right) scale of both axes.

In Figure 4, the goodness-of-fit plots obtained from the final population PK model in linear (left) and logarithmic (right) scale are shown. The upper panel presents the population predictions *vs* the observed concentrations. Especially the data points in the low region were uniformly spread around the line of unity with a slight underprediction in the higher region. Examining the lower panel with individual predicted *vs* observed concentrations, those in the higher region were more uniformly scattered, and the lower concentrations were closer to the line of unity. Overall, the plots indicate that the study data were sufficiently well described by the developed model.

## DISCUSSION AND CONCLUSION

In this study, a population PK analysis was performed for the humanised mAb matuzumab directed against the EGFR with data from three phase I studies. The model was developed using over 1200 serum concentration data points from 90 cancer patients with widely differing characteristics and multiple dosing regimens.

The structural model comprised two compartments with two elimination pathways from the central compartment, one linear and one nonlinear (Michaelis–Menten). Nonlinear PK behaviour has also been reported for other mAbs, such as sibrotuzumab and clenoliximab (Mould *et al*, 1999; Kloft *et al*, 2004). It has also been investigated in addition to the linear elimination for rituximab, but the model did not perform significantly better than the simple linear model. However, goodness-of-fit plots suggest a misspecification for high concentrations (Ng *et al*, 2005).

With respect to the structural model, matuzumab was initially distributed to a restricetd central volume of distribution of 3.7 l and an even smaller peripheral volume of distribution of 1.8 l, which indicated that matuzumab was not (largely) distributed apart from serum volume. Beside the low peripheral volume, the intercompartmental clearance *Q* also indicated a limited distribution, which was consistent with the behaviour of endogenous IgG immunoglobulins (Morell *et al*, 1970; Koleba and Ensom, 2006; Kuester and Kloft, 2006). In total, matuzumab showed similar PK characteristics (clearance and volumes of distribution) to other therapeutic mAbs following intravenous administration (Mould *et al*, 1999; Kovarik *et al*, 2001; Bruno *et al*, 2005).

Three components of random variability (interindividual, interoccasional and residual) were implemented into the matuzumab model. With the relatively small residual variability (13.4% CV for the proportional part and a fixed additive error of 0.312 mg l^−1^), it can be suggested that the developed model possess reasonably high predictability. IOV has been rarely investigated in mAb research, but in more recent population PK analysis, it has been included to improve the model (Kloft *et al*, 2004; Fang *et al*, 2007). The estimated IOV of matuzumab (23% CV; RSE, 13%) was in the range or slightly higher than that for other immunologicals: sibrotuzumab, humanised antibody HuCC49CH2 and etanercept showed 13, 11 and 28% CV, as well as RSE imprecisions of 25 and 102% (not reported for etanercept), respectively (Lee *et al*, 2003; Kloft *et al*, 2004; Fang *et al*, 2007). The importance of implementing IOV in population PK analysis has been demonstrated (Karlsson and Sheiner, 1993) and investigation of IOV avoids biased population parameter estimates.

The aim of building the covariate model was to find patient- or study-specific characteristics, which could explain and thus reduce the variability of the base model. The inclusion of the covariates WT on *V*_1_ and WT on CLL showed a significant improvement of the model, which could be seen by the reduction of the IIV on CLL by approximately 25%. As no difference was observed between patients of either sex and between matuzumab as a single agent and in combination with gemcitabine, no effect of sex and gemcitabine on the PK of matuzumab might be assumed. Additionally, kidney and liver functions do not seem to influence the PK. These results are in good agreement with investigations of other mAbs. The population PK analysis for trastuzumab showed a significant influence of WT on *V*_1_ but was not considered clinically relevant (Bruno *et al*, 2005). Similar results have been reported for the chimeric antibody basiliximab (Kovarik *et al*, 2001). The incorporation of WT on *V*_1_ for golimumab, a fully human mAb, significantly improved the model (Zhou *et al*, 2007).

Future research could attempt to explain the mechanism of nonlinearity in the PK. An appealing approach might present physiologically based modelling including processes, such as receptor internalisation. It has been demonstrated that binding of a mAb to the neonatal Fc receptor (FcRn) and internalisation of the resulting complex results in the long serum half-life of the antibody (Brambell *et al*, 1964; Ghetie *et al*, 1997). Comparing a modified therapeutic antibody (different Fc part) with its original antibody, the resulting half-life for the modified antibody was shorter. It could be assumed that the modified antibody might less be able to bind to the FcRn and thus, was less protected from elimination (Fang *et al*, 2007).

The overall aim of this final model could be, when correlated to PD or efficacy data, to serve as a tool to guide selection of optimal dose regimens for matuzumab, a highly promising ‘targeted’ cancer therapy. From the performed covariate analysis, it should already be recognised that the results do not suggest dose adjustments for sex, age or organ functions such as liver or kidney. For future studies, the identification of molecular tumour markers are considered as being essential prerequisites to be linked to the PK results in a pharmacokinetic/pharmacodynamic model. This could lead to a better prediction of the response for this new class agent and maximise the patient's benefit.

## Figures and Tables

**Table 1:** Study characteristics with matuzumab given as multiple 1 h i.v. infusions ranging from 400 to 2000 mg, q1w–q3w

**Study**	**Tumour entity**	**Dose regimen**	**Planned sampling times^a^**	**No. of subjects**
1	Advanced pancreatic cancer	400 mg q1w; 800 mg q2w; 800 mg q1w	Baseline, 1, 2, 5, 48, 96, 168, 672, 673, 674, 677, 720, 840^b^	17
2	Various advanced cancer (mainly colon/rectum cancer)	1200 mg q1w, q2w, q3w; 400 mg q3w; 800 mg q3w; 1600 mg q3w	For q1w: baseline, 1, 2, 5, 48, 96, 168^b^; for q2w: baseline, 1, 2, 5, 48, 96, 168, 336^b^; for q3w: baseline, 1, 2, 5, 48, 168, 336, 504, 505, 506, 509, 552, 672, 840, 1008^b^	51
3	Various advanced cancer (mainly colon/rectum cancer)	400 mg q1w; 800 mg q1w; 1200 mg q1w; 1600 mg q1w; 2000 mg q1w (from week2: 1600 mg)	Baseline, 1, 2, 5, 24, 72, 96, 168, 504, 505, 506, 509, 528, 576, 600, 672^b^	22

a Relative time elapsed after start of first infusion, in hours.

b Before and after several other infusions peak and trough concentrations were analysed.

**Table 2:** Characteristics of the study population (ID=subject) including number or median, range and number of missings

	**Study 1**	**Study 2**	**Study 3**	**Total**	**Missings**
Number of IDs, (male/female)	17 (9/8)	51 (33/18)	22 (11/11)	90 (53/37)	0
Age (years), median (min–max.)	65 (40–82)	57 (29–78)	58 (30–71)	60 (29–82)	0
Height (cm), median (min–max)	168 (156–183)	169 (143–198)	170 (150–184)	169 (143–198)	3
Weight (kg), median (min–max)	68 (48–81)	71 (47–125)	72 (44–98)	71 (44–125)	3
Body mass index (kg m^−2^), median (min–max)	24.7 (17.0–30.7)	25.8 (20.1–37.0)	24.3 (15.9–33.9)	24.9 (15.9–37.0)	4
Body surface area (m^2^), median (min–max)	1.77 (1.51–2.01)	1.82 (1.34–2.59)	1.85 (1.44–2.16)	1.82 (1.34–2.59)	4
Creatinine clearance (ml min^−1^), median (min–max)	104 (71–480^a^)	83 (47–180)	108 (47–226)	91 (41–480^a^)	3
Alkaline phosphatase (U l^−1^), median (min–max)	190 (118–1026)	156 (40–403)	235 (96–1309)	171 (40–1309)	0
Lactate dehydrogenase (U l^−1^), median (min–max)	171 (117–926)	542 (305–8041)	187 (76–3243)	426 (76–8041)	0
Study centre number (number of IDs)	1 2 (8) (9)	3 (51)	4 (22)	1 2 3 4 (8)(9)(51) (22)	0
Combination chemotherapy, number of IDs	17	0	0	17	0

a Spurious value in original data set, but without influence on population pharmacokinetic analysis.

**Table 3:**
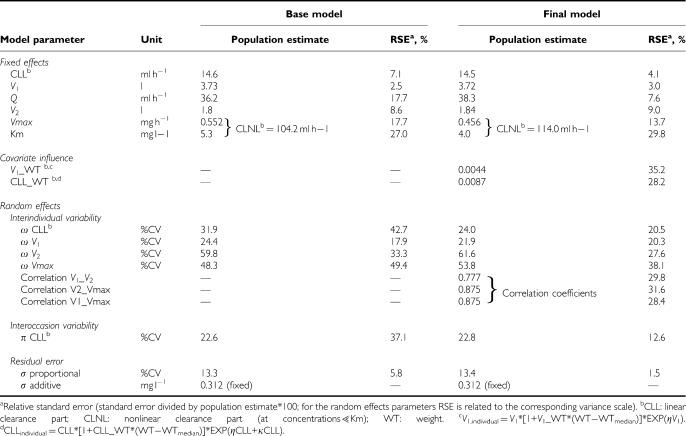
Population pharmacokinetic estimates of matuzumab obtained from the base and the final model
